# SOX9 directly Regulates CTGF/CCN2 Transcription in Growth Plate Chondrocytes and in Nucleus Pulposus Cells of Intervertebral Disc

**DOI:** 10.1038/srep29916

**Published:** 2016-07-20

**Authors:** Chun-do Oh, Hideyo Yasuda, Weiwei Zhao, Stephen P. Henry, Zhaoping Zhang, Ming Xue, Benoit de Crombrugghe, Di Chen

**Affiliations:** 1 Department of Biochemistry, Rush University Medical Center, Chicago, IL 60612, USA; 2 Department of Genetics, The University of Texas, M.D. Anderson Cancer Center, 1515 Holcombe Blvd., Houston, TX 77030, USA; 3 Department of Orthopaedics & Traumatology, Li Ka Shing Faculty of Medicine, The University of Hong Kong, Hong Kong, China

## Abstract

Several lines of evidence indicate that connective tissue growth factor (CTGF/CCN2) stimulates chondrocyte proliferation and maturation. Given the fact that SOX9 is essential for several steps of the chondrocyte differentiation pathway, we asked whether *Ctgf* (*Ccn2*) is the direct target gene of SOX9. We found that *Ctgf* mRNA was down-regulated in primary sternal chondrocytes from *Sox9*^*flox/flox*^ mice infected with Ad-CMV*-Cre.* We performed ChIP-on-chip assay using anti-SOX9 antibody, covering the *Ctgf* gene from 15 kb upstream of its 5′-end to 10 kb downstream of its 3′-end to determine SOX9 interaction site. One high-affinity interaction site was identified in the *Ctgf* proximal promoter by ChIP-on-chip assay. An important SOX9 regulatory element was found to be located in −70/−64 region of the *Ctgf* promoter. We found the same site for SOX9 binding to the *Ctgf* promoter in nucleus pulposus (NP) cells. The loss of *Sox9* in growth plate chondrocytes in knee joint and in NP cells in intervertebral disc led to the decrease in CTGF expression. We suggest that *Ctgf* is the direct target gene of SOX9 in chondrocytes and NP cells. Our study establishes a strong link between two regulatory molecules that have a major role in cartilaginous tissues.

Endochondral ossification is an essential process that occurs during the development of the mammalian skeleton^1^. In this process, the condensed mesenchymal cells differentiate, through several steps, into hypertrophic chondrocytes^2^. The ossification is initiated within the cartilage template after calcified cartilage is removed and replaced by osteoblast progenitor cells. Connective tissue growth factor (CTGF)/CCN2 is a member of the CCN family, which contains four common domains, including an insulin-like growth factor binding protein domain, a von Willebrand type C repeat, a thrombospondin type I repeat, and a C2 terminal cysteine-knot^3^. CTGF is a secreted protein and likely functions by binding to a trans-membrane receptor based on its structure^3^. CTGF has been shown to have multiple functions and is involved in wound-healing^4^ and skeletal development^5^,^6^. *Ctgf* knockout (KO) mice die right after birth owing to respiratory failure caused by impaired endochondral ossification, indicating its essential role in normal skeletal development^7^. Furthermore, during chondrocyte differentiation, CTGF has been shown to be expressed strongly in hypertrophic zone^7^. The promoter region of *Ctgf* contains a TATA box and binding sites for several transcriptional activators or modulators, such as a binding site for Smad and a TGF-β responsive element, through which TGF-β regulates *Ctgf* expression^8^,^9^,^10^. Furthermore matrix metalloproteinase 3 (MMP-3), a well-known secretory endopeptidase, has also been shown to function as a transcriptional activator of *Ctgf* by binding to an enhancer region of the *Ctgf* gene in chondrocytes^11^.

SOX (SRY-related HMG box) proteins have critical functions in a number of developmental processes. SOX9 plays an essential role in determining the fate of several cell types^12^,^13^,^14^,^15^ and has been considered as a master regulator of chondrocyte development^16^. *Sox9* mutations were found in the campomelic dysplasia, an autosomal dominant human disease, which severely affects the skeletal development^17^,^18^. In addition, mice with heterozygous *Sox9* mutations display a similar skeletal phenotype^19^,^20^. SOX9 has a Sry-related high-mobility group-box DNA binding domain that preferentially binds the AGAACAATGG sequence *in vitro*^21^. Furthermore, genetic studies in mice and *in vitro* studies of chromatin immunoprecipitation (ChIP)-on-chip analysis, reporter assay, and electrophoretic mobility shift assay (EMSA) have shown that SOX9 is required for the expression of typical cartilage matrix protein-coding genes (*Col2a1, Col11a2*, *Aggrecan, CD-rap,* etc.) which serve as direct targets of SOX9^22^,^23^,^24^,^25^,^26^,^27^. Since SOX9 is a master regulator of chondrocyte differentiation, we asked whether CTGF, which has a critical role in cartilage development, is a direct target of SOX9. To test this, we depleted *Sox9* in chondrocytes, analyzed the interaction of SOX9 in the chromatin of the *Ctgf* gene by ChIP-on-chip assay, and measured *Ctgf* transcription using the reporter assay. Our results showed that *Ctgf* is the direct target of SOX9 in chondrocytes.

Furthermore, the *Ctgf* has been shown to stimulate extracellular matrix production by nucleus pulposus (NP) cells^28^ and TGF-β, through Smad3 and AP1, serves as a positive regulator of *Ctgf* expression in NP cells^29^. However, it is not known if *Ctgf* expression is regulated by SOX9 in disc tissues. Thus, we investigated SOX9-binding sites in the *Ctgf* promoter region using ChIP and EMSA techniques in NP cells. We showed that SOX9 binds to the *Ctgf* promoter in NP cells and loss of CTGF expression was detected in *Sox9* deleted disc cells.

## Results

### Deletion of Sox9 in primary sternal chondrocytes reduces Ctgf mRNA and protein expression

**Figure 1:**
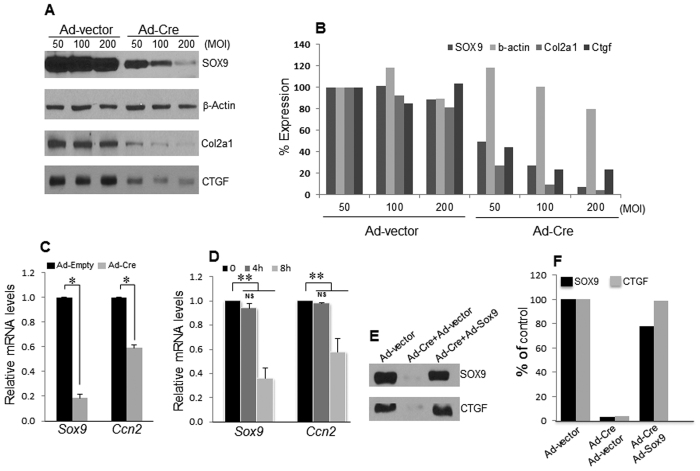
CTGF expression was reduced in Sox9-deficient primary sternal chondrocytes. (**A**) Protein levels in the *Sox9*-deficient primary sternal chondrocytes. Primary rib chondrocytes were prepared from *Sox9*^*floxflox*^ mice, cultured, and infected with different concentrations of Ad-CMV-*Cre*. Seventy-two hours after infection, the cells were harvested and the levels of the various proteins were analyzed by Western blotting. (**B**) Amounts of protein levels with different concentrations of infection of Ad-CMV-*Cre* were measured using a phosphoimager and plotted as percentage shown in (**A**). (**C**) Differences in the mRNA levels of *Ctgf* and *Sox9* between Ad-CMV-*Cre* and an empty vector (Ad-CMV-Null)-infected cells. The rib chondrocytes isolated from *Sox9*^*floxflox*^ mice were infected with Ad-CMV-*Cre* or Ad-CMV-Null at 200 moi. The total RNA was extracted from the cell cultures and the expression levels were measured. The differences in the Ct values (delta Ct) of the mRNA levels were analyzed in the Ad-CMV-*Cre-* and Ad-CMV-Null-treated cells. The data were normalized to the expression levels of the housekeeping gene *Gapdh*. (**D**) Primary rib chondrocytes were also prepared from *Sox9*^*Col2ER*^ conditional KO mice at postnatal day 4. The rib chondrocytes were treated with 4-Hydroxytamoxifen (4-OH-TM, 1 μM) and the total RNA was extracted at indicated time point and the expression levels were measured. (**E**) The primary chondrocytes prepared from *Sox9*^*floxflox*^ mice were infected with Ad-CMV-*Cre* or Ad-CMV-Null at 100 moi. Twenty-four hours after incubation, the cells were infected with Ad-CMV-*Sox9* or Ad-CMV-Null at 100 moi for another 24 h. The expression level of the SOX9 and CTGF proteins in each culture was determined by Western blotting. The CTGF level was decreased by *Sox9* depletion and restored by the expression of Sox9. (**F**) Quantitative densitometry of the protein expressions of the SOX9 and CTGF expression shown in (**E**). Values are means ± SEM; *p < 0.01, **p < 0.05, NS: not significant.

*Sox9*^*flox/flox*^ mice, in which exons 2 and 3 of the *Sox9* gene are flanked by *loxP* sites, have a normal phenotype. Removing the floxed alleles by crossing the mice with *Cre*-deleter leads to severe defects in skeletal^16^. We cultured sternal chondrocytes isolated from *Sox9*^*flox/flox*^ mice and infected with various concentrations of Cre recombinase expressing adenovirus (Ad-CMV-*Cre*) to remove *Sox9* from cultured chondrocytes. Cells infected with empty vector (Ad-CMV-*Null*) were used as controls. Results of Western blotting showed that *Sox9* was efficiently removed in a dose-dependent manner when the cells were infected with Ad-CMV*-Cre* (Fig. 1A). Infecting the cells with a 50- or 200-multiplicity of infection (moi) reduced the SOX9 protein level to <50% or <10% of its initial levels in cells infected with 50- or 200-moi of Ad-CMV*-Null,* respectively. Expression levels of β-actin, which served as an internal control, remain unchanged during the entire experimental period. In contrast, collagen II, which is known to be a direct target of SOX9^27^, was reduced to 25% or <10% of its initial levels with 50- or 200-moi of Ad-CMV-*Cre* infection, respectively. CTGF levels were also decreased in a dose-dependent manner with the increasing concentrations of Ad-CMV*-Cre.* CTGF was reduced to 50% or <30% of its control levels with 50- or 200-moi of Ad-CMV-*Cre* infection, respectively. Quantitative densitometry analysis was shown as plotted graph in Fig. 1B. These findings suggest that SOX9 directly affects the expression of CTGF. Figure 1C showed *Sox9* and *Ctgf* mRNA levels after removal of the *Sox9* gene by Ad-CMV*-Cre* infection. *Sox9* mRNA was decreased 81.6% compared to its control levels. *Ctgf* mRNA was reduced 42.8% compared to the control. To support the hypothesis that SOX9 directly regulates CTGF expression we isolated primary sternal chondrocytes from *Sox9*^*Col2ER*^ mice at postnatal day 4 and treated cells with 1 μM of 4-Hydroxytamoxifen (4-OH-TM) to these cells. *Sox*9 and *Ctgf* mRNA was not significantly changed 4 hours after 4-OH-TM treatment but *Sox9* and *Ctgf* mRNA decreased 64% and 42%, respectively, compared to its control levels 8 hours after 4-OH-TM treatment (Fig. 1D). These results indicate that *Ctgf* expression could be down-regulated when *Sox9* was deleted within 4 hour period. Next we examined whether CTGF levels are restored when the SOX9 expression is rescued after its deletion by Ad-CMV*-Cre*. Primary sternal chondrocytes were treated with 100-moi Ad-CMV*-Cre* for 24 h, and then the culture was infected with 100-moi Ad-CMV-*Sox9* or Ad-CMV*-Null* for additional 24 hours. When the cells were infected with Ad-CMV-*Sox9*, SOX9 levels were returned to nearly its initial levels and CTGF levels were also restored to its initial levels (Fig. 1E,F). These results are consistent with the hypothesis that SOX9 directly regulates the *Ctgf* expression.

### Ctgf gene contains a SOX9 interaction site in chondrocytes

**Figure 2:**
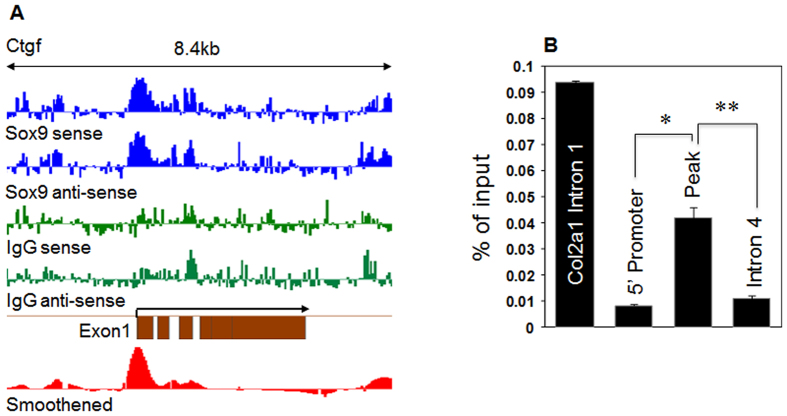
Identification of the SOX9 interaction site in the *Ctgf* gene. (**A**) ChIP-on-chip using aSOX9 antibody showed a specific hybridization peak near the transcription initiation site in the *Ctgf* gene. Exons are shown as solid bars. (**B**) Validation of the hybridization peak shown in (**A**) using ChIP-qPCR. QPCR was performed using primers specific for different regions of *Ctgf* and ChIP-DNA as the template. Primers for the SOX9 interaction site in *Col2a1* intron1 were used as a positive control. The other primers used were specific for the peak in (**A**) (−33/+48), the *Ctgf* 5′-promoter region (−743/−676) and intron 4 (+1450/+1530). The Y axis showed the level of ChIP DNA by the percentage of input DNA. Values are means ± SEM; *p < 0.01, **p < 0.001.

If *Ctgf* is a direct target of SOX9, we expect to identify a SOX9 interaction site within the *Ctgf* gene. To assess this, we previously performed a ChIP-on-chip experiment using an anti-SOX9 antibody^27^. The experiment was performed using a rat chondrosarcoma (RCS) cell line that retains many characteristics of chondrocytes^30^. The ChIP DNA precipitated with the anti-SOX9 antibody or with non-specific IgG was hybridized to a custom-made high-density microarray, which covered the *Ctgf* gene from 15 kb upstream of its 5′-end to the 10 kb downstream of its 3′-end. In this experiment we demonstrated that *Ctgf* gene contains a high affinity SOX9 interaction site in its promoter region^27^. We then further determined the precise position of the hybridization peak. In this experiment the IgG hybridization value was subtracted from the hybridization value obtained with the SOX9 antibody. One high-affinity SOX9 interaction site was detected in ChIP-on-chip analysis using both sense and anti-sense DNA probes. The peak of the SOX9-interaction curve was located close to the transcription initiation site (+1) (Fig. 2A).

To validate the SOX9 interaction site obtained from the ChIP-on-chip experiment, qPCR was performed using the anti-SOX9-ChIP DNA as a template. We observed a strong amplification region in the *Ctgf* gene near the peak detected by ChIP-on-chip. The well-characterized SOX9 interaction site in intron 1 of the *Col2a1* gene was used as a positive control, and the amplicon was clearly detected in this region using the ChIP DNA as a template. The level of the amplification of the *Col2a1* intron was more than twice as that detected in the *Ctgf* peak near its transcriptional initiation site (−33/+48). By contrast, the DNA segments in the 5′ promoter (−743/−676) and intron 4 (+1450/+1530), where a SOX9 interaction site was not detected by ChIP-on-chip, were not amplified when anti-SOX9-ChIP DNA was used as a template (Fig. 2B). These results suggest that a SOX9 interaction site is located near the transcription initiation site of the *Ctgf* gene.

### Identification of the SOX9 binding element necessary for Ctgf transcription

**Figure 3:**
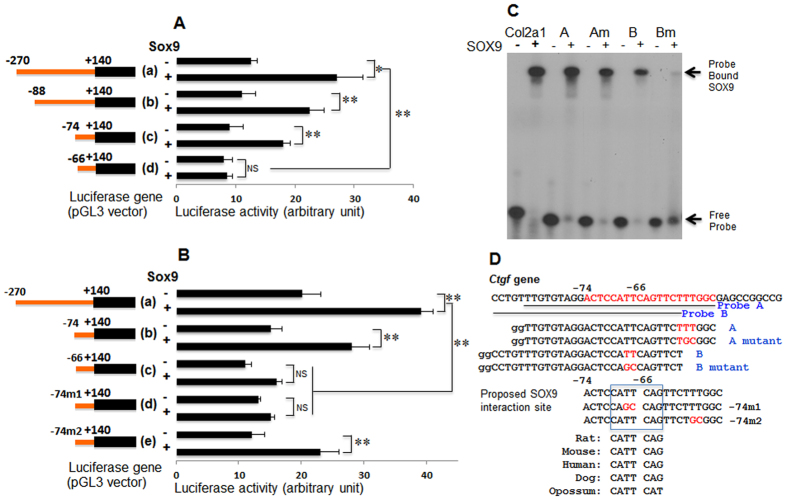
Stimulation of *Ctgf* transcription by SOX9 and validation of the SOX9 interaction site by EMSA. (**A**) Schematic representation of the constructs used is shown on the left. The numbers correspond to the nucleotides with respect to the transcription start site. 293T cells were transfected with different reports construct (200 ng) with or without *Sox9* plasmid (600 ng) and with 5 ng of CMV-*renilla* luciferase plasmid, which served as an internal control for transfection efficiency. The values shown are the *firefly* luciferase activity of the construct normalized to the *renilla* luciferase activity. (**B**) The constructs (d) and (e) have mutations in the −74/+140 region. The sequence of part of this region is shown in (**D**); the mutated sites are denoted in red. On the right, the luciferase activity of each construct is shown. The values represent the *firefly* luciferase activity of the construct normalized to the *renilla* luciferase activity. (**C**) EMSA was performed using the SOX9 interaction site in the *Ctgf* promoter that we have identified. (**D**) The sequences of the mutated and control probes are shown in (**D**). 20 ng of SOX9 were used in each lane. The ^32^P-labeled probes bound to SOX9 (a.a. 1–300) have less mobility in the gel than the free probes. Data represent mean ± SEM; *p < 0.001, **p < 0.05; NS: not significant.

The results of the ChIP-on-chip experiment suggest that the SOX9 interaction site in the *Ctgf* gene was located near the transcription initiation site. Thus, several reporter constructs that contain different lengths of the *Ctgf* 5′-promoter region and a part of exon 1 were examined to determine which fragment of the *Ctgf* promoter is responsive to SOX9 stimulation in HEK-293T (293T) cells. The longest construct is the −270/+140 fragment of the *Ctgf* promoter fused to the luciferase cDNA (pGL3 vector). SOX9 stimulated the reporter activity by 2.0-fold in −270/+140-Luc reporter (Fig. 3A). The construct of −74/+140-Luc was also activated by SOX9 (Fig. 3A,c), but the −66/+140-Luc construct was not (Fig. 3A,d). Thus, an essential sequence for the SOX9-dependent activation of the reporter seems to be located in the −74/−66 region. This sequence is not the SOX9 consensus binding motif, C(A/T)TTG(A/T)(A/T), but five (CATTcAg) of the seven bases in the −70/−64 region match the SOX9 consensus motif. To confirm if this sequence is essential for SOX9 binding, we mutated the potentially important nucleotides in this motif, and the mutant constructs were examined using the reporter assay (Fig. 3B). We found that mutation of TT to GC (−67 and−68) abrogated the SOX9-dependent enhancement of the luciferase reporter activity (Fig. 3B,d). The other mutations at −59 and −58, which also contain a similar potential SOX9 interaction motif, had only minor effect on the SOX9-induced reporter activity (Fig. 3B,e). Taken together, these data suggest that −70/−64 of the *Ctgf* promoter contains a functional SOX9 interaction site. To further confirm that −70/−64 of *Ctgf* indeed contains a SOX9 interaction site and SOX9 binds to its responsive element in this region, an EMSA was performed using a probe corresponding to the putative SOX9 interaction site. SOX9 clearly bound to its binding sites in *Col2a1* intron 1 (positive control, Fig. 3C, lanes 1 and 2). Two *Ctgf* promoter fragments containing the putative SOX9 interaction site were examined for their ability to bind SOX9. SOX9 bound to both fragments (probes A (−83/−55) and B (−88/−60) containing −70/−64 of the *Ctgf* binding site (Fig. 3C, lanes 4 and 8). To confirm SOX9-DNA interactions with both fragments, we have performed supershift assay using the SOX9 antibody and the results showed the clear binding consistent with Fig. 3C (Fig. S1). We then examined the binding of SOX9 to the probes with mutations in the putative SOX9 interaction site. SOX9 still bound to the Probe A mutant containing a TT-to-GC (−59 and −58) mutation that was outside the putative interaction site, though the binding was slightly reduced (Fig. 3C, lanes 5 and 6). In contrast, SOX9 bound weakly to the Probe B mutant containing the TT-to-GC mutation (−68 and −67) in the putative SOX9 interaction site (Fig. 3C, lanes 9 and 10). These data are consistent with the results obtained from the reporter assay, showing that CATTCAG (−70/−64) is the SOX9 interaction site responsible for the transcriptional activation of the *Ctgf* promoter by SOX9. The location of the mutation used in luciferase reporter and EMSA experiment is presented in Fig. 3D.

### Monomeric SOX9 binding to the Ctgf promoter

**Figure 4:**
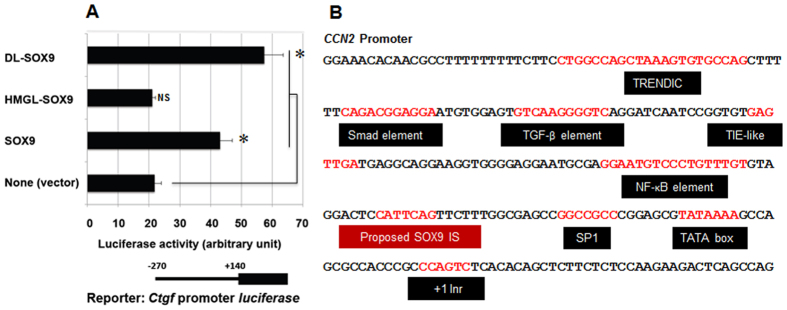
Stimulation of Ctgf transcription by monomeric SOX9. (**A**) Each mutant construct of *Sox9* that does not form dimer (DL (dimer less)-SOX9) or does not have DNA binding domain (HMGL (HMG domain-less)-SOX9) was transfected into 293T cells as shown in Fig. 3. The construct that had −270/+140 of *Ctgf* promoter fused to pGL3 vector was used as the reporter. The DL-SOX9 enhanced the reporter activity but HMGL-SOX9 did not. (**B**) Rat *Ctgf* gene promoter with major regulatory binding elements and proposed SOX9 interaction site are shown in (**B**). Values are means ± SEM; *p < 0.01; NS: not significant.

SOX9 forms a dimer with pairs of sites arranged in an inverted repeat configuration^31^ and SOX9 also binds as a monomer to the regulatory region of the sex-determining gene *SF1*^32^. Therefore we tried to confirm whether SOX9 dimerization is required for its binding to the *Ctgf* promoter using a monomeric SOX9 mutant that does not form a dimer in the reporter assay. In Fig. 4A, the monomeric SOX9 (dimer less SOX9, DL-SOX9) did enhance the reporter activity but the HMG-less SOX9 (HMGL-SOX9) did not. In Fig. 4B, our proposed SOX9 binding site in the *Ctgf* promoter was shown along with major regulatory binding elements as previously reported^33^.

### The Ctgf gene contains a SOX9 interaction site in rat NP cells

**Figure 5:**
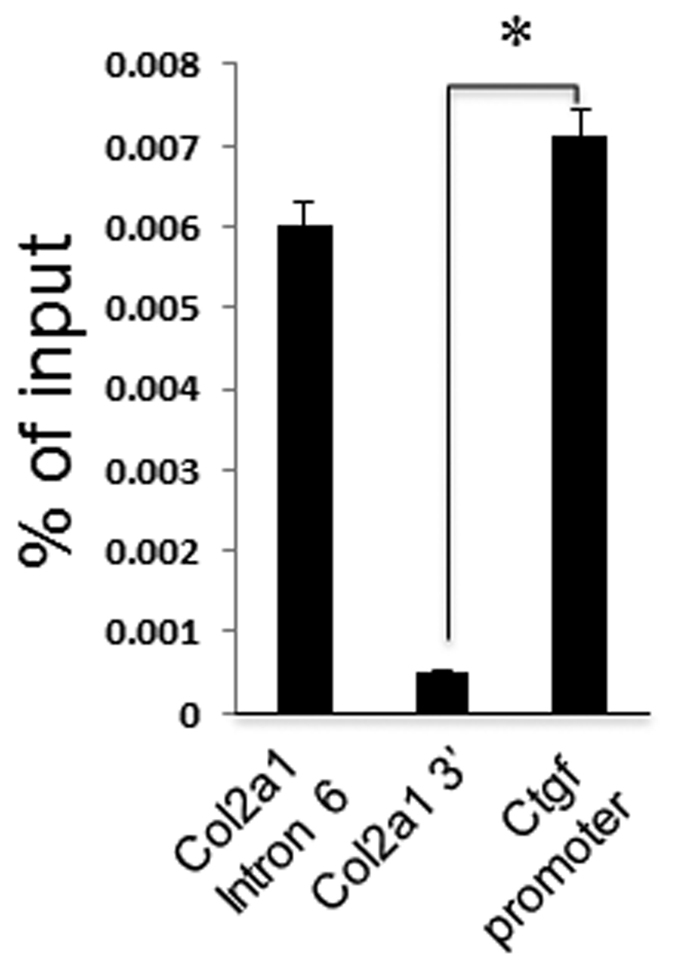
Identification of the SOX9 interaction site in the *Ctgf* gene in NP cells. Validation of the hybridization peak shown in (**A**) using ChIP-qPCR. QPCR was performed using primers specific for different regions of the *Ctgf* gene and ChIP-DNA as the template. Primers for the SOX9 interaction site in *Col2a1* intron 6 were used as a positive control. The primers used were specific for the peak in (**A**) (−33/+48). The Y axis showed the level of ChIP DNA by the percentage of input DNA. Values are means ± SEM; *p < 0.001.

The homeostasis of the intervertebral disc (IVD) was severely disrupted upon the depletion of *Sox9*, which was observed in histology of the IVD two weeks after tamoxifen injection to *Sox9*^*Agc1ER*^ mice^34^. In addition, embryonic discs from CCN2 null mice showed decreased Safranin O and aggrecan staining compared to discs of wild type mice and a significant decrease in the expression of aggrecan in *Ccn2* silenced human NP cells^1^. Therefore, we examined if SOX9 regulates *Ctgf* transcription in NP cells of disc tissues. Results of SOX9-ChIP-qPCR experiments showed that levels of the amplification detected in the *Ctgf* peak near its transcription initiation site (−33/+48) was more than that detected in the well-characterized SOX9 interaction site in the *Col2a1* intron 6 used as a positive control. In contrast, the DNA segments in the 3′ site of *Col2a1* used as a negative control were not amplified when anti-SOX9-ChIP DNA was used as a template (Fig. 5A). These results suggest that a SOX9 interaction site is located near the transcription initiation site of the *Ctgf* promoter in rat NP cells. To validate that *Ctgf* promoter indeed contains a SOX9 interaction site, an EMSA was performed using a probe corresponding to the putative SOX9 interaction site. SOX9 clearly bound to its binding sites in *Col2a1* intron 1 (positive control; Fig. 5B, lanes 4–6). *Ctgf* promoter fragments containing the putative SOX9 interaction site were examined for their ability to bind SOX9. SOX9 bound to both fragments in NP cells (probes containing −70/−64 of the *Ctgf* promoter) (Fig. 5B, lanes 1–3).

### CTGF expression is reduced in growth plate of knee joint and NP cells of disc tissues in Sox9 conditional KO mice

**Figure 6:**
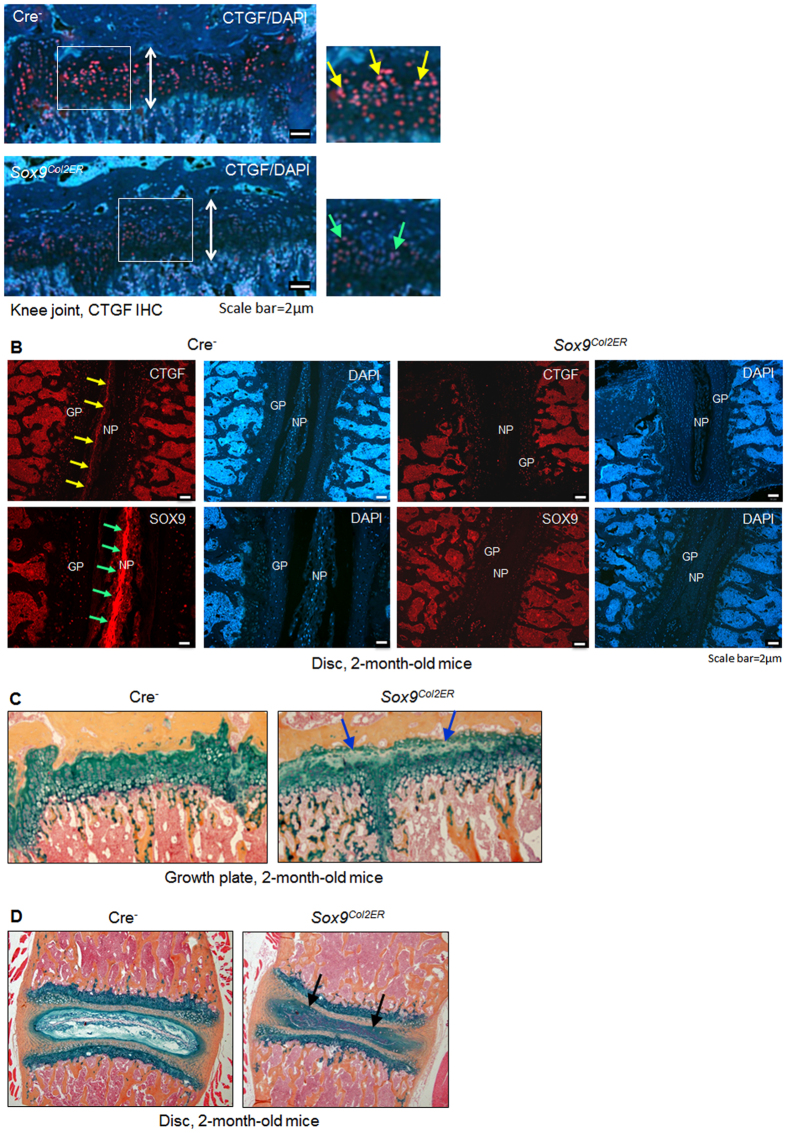
CTGF and SOX9 expression in knee joint and intervertebral disc. (**A**) CTGF and SOX9 expression in growth plate of knee joints. Growth plate from 2-month-old knee joint was stained with CTGF in Cre^−^ (Control) (yellow arrows) and *Sox9*^*Col2ER*^ conditional KO mice (green arrows) injected with tamoxifen at postnatal day 20. CTGF expression was significant reduced in *Sox9* conditional KO mice. (**B**) CTGF (yellow arrows) and SOX9 (green arrows) expression in growth plate (GP) and nucleus pulposus (NP) cells of disc tissues. Disc tissues were stained with CTGF or SOX9 in Cre^−*x*^ (Control) and *Sox9*^*Col2ER*^ conditional KO mice injected with tamoxifen at postnatal day 20. CTGF was expressed in GP and NP cells in control mice. Deletion of *Sox9* in *Col2a1*-expressing disc cells exhibits decrease of CTGF expression. All sections were counterstained with DAPI (blue). (**C**,**D**) Histologic analysis in growth plate of knee joint and disc. Growth plate from 2 months old knee joint (**C**) and lumbar disc (**D**) were performed on paraffin embedded slides and stained with Alcian blue/hematoxylin and eosin (AB/H&E) in *Sox9*^*floxflox*^ (Control) and *Sox9*^*Col2ER*^ conditional KO mice injected with tamoxifen at postnatal day 20. Histologic results demonstrated the loss of proteoglycan in growh plate (blue arrows) and shrunk NP tissue (black arrows) of *Sox9*^*Col2ER*^ conditional KO mice.

We next examined CTGF expression in growth plate and intervertebral disc in 2-month-old *Sox9*^*Col2ER*^ mice which were injected with tamoxifen at postnatal day 20. The CTGF was highly expressed in proliferating, pre-hypertrophic and hypertrophic chondrocytes in control mice but was weakly expressed in those cells in knee joint of *Sox9* conditional KO mice (Fig. 6A). The *Sox9* deletion was confirmed by SOX9 immunostaining. SOX9 was highly expressed in proliferating and pre-hypertrophic chondrocytes but was weakly expressed in *Sox9*^*Col2ER*^ conditional KO mice (Fig. S2). Similarly, CTGF and SOX9 were highly expressed in NP cells and growth plate (GP) chondrocytes of disc tissues in control mice (Fig. 6B) but were weakly expressed in those cells of *Sox9*^*Col2ER*^ conditional KO mice (Fig. 6B), indicating that the decrease of SOX9 expression in disc tissues correlates with the reduction of CTGF expression in growth plate chondrocytes of knee joints of *Sox9* conditional KO mice. Consistent with the reduced SOX9 and CTGF expression, histologic analysis showed reduced proteoglycan levels in growth plate of knee joint (Fig. 6C) and loss of proteoglycan and water retention in NP of disc tissue (Fig. 6D).

## Discussion

In this study we showed that *Ctgf*/*Ccn2* is regulated by SOX9 through direct binding to the −74/−66 promoter region. Expression levels of CTGF were markedly reduced in *Sox9*-deficient rib chondrocytes and restoring the SOX9 levels with Ad-CMV-*Sox9* preserved CTGF levels. We also showed that removal of *Sox9* mRNA reduced the *Ctgf* mRNA levels by both qPCR and RNA-Seq approaches in chondrocyte cultures (Fig. S3). Furthermore, in the intervertebral disc of *Sox9*^*flox/flox*^ mice, where the expression of a number of matrix proteins that are direct targets of SOX9 parallels to those in chondrocytes^33^, the Sox9 mRNA level in the *Sox9*-deficient discs was reduced 90% and *Ctgf* mRNA levels were reduced 91% compared to its control levels by qPCR^34^. In this experiment the 8-week-old *Sox9*-deficient mice were obtained by crossing *Sox9*^*flox*/*flox*^ mice with *Agc1-CreER*^*T2*^ mice and the treatment with tamoxifen was performed at 7-week-old mice^34^. These results indicate that SOX9 also regulates the expression of *Ctgf* in intervertebral disc tissues.

Huang *et al*., showed that SOX9 represses *Ctgf* induction through binding to a SOX9-TCF (LEF) consensus binding site, AACAAAG around 0.5-kb upstream from its transcription initiation site in osteo-chondro progenitor cells and that in hypertrophic chondrocyte this site is occupied by TCF (LEF) where *Ctgf* genes were up-regulated^35^. However, in our anti-SOX9 ChIP-on-chip experiment, which has been proven to work efficiently^27^, no SOX9 interacting site was detected around this region (Fig. 2). Instead, we found a SOX9 interaction site located in the proximal promoter region of the *Ctgf* gene which was activated by SOX9. In the study reported by Huang *et al*., four conserved consensus binding sites for TCF·LEF were identified via TRANSFAC analysis. Given the fact that some TCF·LEF consensus binding sites are also consensus sites for SOX9, EMSA assay was performed, and it was demonstrated that either SOX9 or TCF·LEF factors could bind to the TCF·LEF site at −443 bp. In our study, one high-affinity SOX9 binding site was detected in the *Ctgf* promoter using ChIP-on-chip analysis. Furthermore, Huang *et al*. suggested a stage-specific occupancy of the TCF·LEF·SOX9 in undifferentiated and hyperthrophic ATDC5. Whereas, RCS cells, used in our study, displayed a stable early differentiated chondrocyte phenotype. It seems that differences of cell types and differentiation stages may contribute to the discrepancy.

The *Ctgf* gene has typical core promoter elements, including a TATA box and binding sites for several transcriptional activators or modulators. Others have shown that the expression of *Ctgf* is enhanced by TGF-β signaling through a TGF-β responsive element^8^. In addition, sphingosine 1-phosphate (S1P) that requires a separate Smad element for its activity also enhances *Ctgf* expression^36^. The *Ctgf* promoter region also contains a S1P binding site as well as an enhancer to which MMP-3 binds and enhances its transcription^11^. Although many factors could potentially regulate *Ctgf* transcription, when we deleted *Sox9* in chondrocytes, both mRNA and protein levels of CTGF decreased markedly. CTGF levels were restored when SOX9 levels were preserved. These results suggest that SOX9 is a major transcription factor regulating *Ctgf* expression in chondrocytes. SOX9 functions as a master regulator of several steps in chondrocyte differentiation. It binds to specific sites in its target genes and promotes their transcriptional activities. The SOX9 binding sites in several genes encoding for cartilage matrix proteins have been proposed to form an inverted repeat^31^,^32^,^37^. Considering the data of the reporter assay and EMSA, we proposed the CATTCAG motif in the *Ctgf* promoter is a true SOX9 binding site. When we surveyed the Sry consensus binding site in the *Ctgf* promoter using TFSEARCH data base ( http://www.cbrc.jp/research/db/TFSEARCH.html), a little longer fragment than that, CATTCAGTTC, was detected^38^. In both cases the promoter region of *Ctgf* appears to contain a single binding site for SOX9 (Fig. 3). SOX9 can form a transcription activation complex with several proteins and has been shown to bind several transcription factors and transcriptional co-activators, including LSOX5, SOX6^39^, TIP60^40^, p300/CBP^41^, and Znf219^42^, but the detail structure of the transcriptional activation complex remains unknown.

More recently, the role of CTGF in disc tissues was examined but its function was relatively less known. Tran *et al*. showed that hypoxia regulates CCN2 in a HIF-1α dependent manner in NP cells; whereas CCN2 controls HIF-1α levels, indicating that a negative feedback loop exists in NP cells^43^. In addition, the same group found that IL-1β and TNFα suppress CCN2 expression through the NF-κB signaling in NP cells^44^. Based on these findings, we examined whether *Ctgf* could be regulated by SOX9 in disc tissues. Interestingly SOX9 regulates *Ctgf* expression through its binding site located in the proximal promoter region of the *Ctgf* gene in NP cells, demonstrated by ChIP-qPCR (Fig. 5). Furthermore the CTGF expression was decreased in growth plate of knee joint and NP cells of disc tissues from *Sox9*^*Col2ER*^ conditional KO mice (Fig. 6B).

In summary, we have shown that *Ctgf* expression is directly regulated by the transcription factor SOX9 in chondrocytes and the SOX9 binding site was also detected in NP cells. Our studies underline the importance of SOX9 in regulation of chondrocyte differentiation through CTGF, an indispensable factor in cartilage development. The detail mechanism by which SOX9 controls *Ctgf* expression in NP cells needs to be further investigated.

## Methods

### Cell culture and adenovirus infection

RCS cells^30^ and 293T cells were cultured at 37 °C in Dulbecco’s modified Eagle’s medium (DMEM) supplemented with 10% fetal bovine serum. Primary sternal chondrocyte cells were cultured as described previously^45^ using 4-day postnatal *Sox9*^*floxflox*^ mice^16^ or *Sox9*^*Col2ER*^ mice. The titers of the adenoviruses prepared by the Genetically Engineered Mouse Core facility at Baylor College of Medicine were obtained by using an Adeno-X rapid titer assay kit (Clontech, Mountain view, CA) according to the manufacturer’s protocol. 1 μM of 4-Hydroxytamoxifen (4-OH-TM, Sigma, St Louis, MO) were treated to cultured cells. Rat NP cells were isolated using a method reported earlier by Im *et al*.^46^. NP cells were maintained in DMEM/Ham’s F-12 (1:1) culture medium with 20% fetal bovine serum (FBS).

### Western blotting and measurement of mRNA expression

Western blotting was performed as described previously. After washing the cells twice with phosphate buffered saline, cell lysates were prepared in RIPA buffer, (10 mM Tri-HCl, pH 7.4, 0.01% sodium dodecyl sulfate (SDS), and 0.1% Nonidet P-40). The proteins in cell lysates were separated by SDS-10% polyacrylamide gel electrophoresis and were transferred to a nitrocellulose membrane. The membranes were treated with the appropriate primary antibody and then with the appropriate secondary antibody labeled with horseradish peroxidase. The primary antibodies used were a SOX9 antibody (Millipore, Billerica, MA), collagen II antibody (Abcam, Cambridge, MA), β-actin antibody (Abcam), and CTGF antibody (Santa Cruz Biotechnology, Santa Cruz, CA). The horseradish peroxidase was detected using the ECL detection kit (Thermo Fisher Scientific (Pierce), Rockford, IL). To measure the mRNA expression level, total RNA was extracted from primary chondrocytes using Trizol reagent (Invitrogen, Carlsbad, CA) according to the manufacturer’s protocol. cDNA was prepared from the mRNA using AMV reverse transcriptase (Invitrogen). Quantitative qPCR was performed using primers specific for each RNA, SYBR Master Mix, and an ABI 7900 real-time PCR system (Applied Biosystems, Foster city, CA ), as described previously^27^. The difference in Ct values (delta Ct) between the Ct value of each sample and that of GAPDH was calculated. The delta Ct value of each gene was then compared to that of each sample.

### ChIP-on-chip and ChIP-qPCR analysis

ChIP was performed according to the previously described method^27^ using a ChIP assay kit (Millipore, Billerica, MA). Briefly, the RCS cells were fixed with formaldehyde, and the chromatin prepared by sonication was treated with rabbit anti-SOX9 antibodies (Millipore, AB5809) or non-specific rabbit IgG antibodies. The resultant DNA fragments were ligated with random oligonucleotides after the DNA was modified with terminal deoxyribonucleotide transferase. The modified anti-SOX9-precipitated and IgG-precipitated DNA fragments were amplified by PCR and further labeled with Cy3 and Cy5, respectively. The chip array was done using the NimbleGen platform (NimbleGen, Madison, WI). The ChIP-qPCR experiments were performed using the SYBR Green PCR Master Mix and an ABI7900HT qPCR system (Applied Biosystems) using ChIP-DNA as a template. The percentage of input was calculated using the Ct value of the input DNA and ChIPDNA. The data were also normalized to the value of the IgG control antibody. The primers used for qPCR are as follows: *Col2a1* intron 1, TGAGGCTTGTTTGCGTTGAG and AGGGCATGGTGACTCAGATG; 5′ promoter (−743 to −676) of *Ctgf*, ACTCCATGCCCAGTCATTGTC and CAGCCCTCTTTATGTAAGGACTTGT; the peak (−33 to +48) of *Ctgf,* GAGTGGGTCTGGCTGAGTCTTC and GCCCGGAGCGTATAAAAGC; and intron 4 (+1450 to +1530) of *Ctgf,* TCTCGCCGCCCTTCTTATTA and AGCATCTCTCATTCTAGCCAGACA.

### Plasmid construction and reporter assay

*Ctgf* fragments containing different portions of its promoter region were prepared by PCR using BacCH230-371G22 from the BACPAC Resource Center (Oakland, CA) as a template. The mutant promoter fragments were prepared by using a site-directed mutation system according to the manufacturer’s protocol (Stratagene, Santa Clara, CA). The PCR-amplified fragments cut with BamH1 and HindIII at their 5′- and 3′-ends, respectively, were cloned into the BglII/HindIII sites of the pGL3 luciferase plasmid (Promega, Madison, WI). The mutants of Sox9 constructs (DL-SOX9 and HMGL-SOX) were shown previously^47^. The sequences of all constructs were verified by DNA sequencing. The reporter assay was done as described previously^27^. Briefly, 1.5 × 10^5^ 293T cells were inoculated into each well of a 12-well plate. Twenty-four hours after incubation, the cells were cotransfected with the appropriate reporter plasmid construct (0.2 μg/well), a SOX9-expressing plasmid (0.6 μg/well), and a CMV-*renilla* luciferase plasmid (5 ng/well), which was used as a transfection control, using Fugene 6 (Clontech, Mountain view, CA). After 36 hr incubation, the luciferase activity was measured using a dual luciferase assay system (Promega). Each value in the reporter assay is presented as the fold-increase in *firefly* luciferase activity per *renilla* luciferase activity units from three independent experiments.

### Electrophoretic mobility shift assay

Electrophoretic mobility shift assays (EMSAs) were performed as described previously^47^ using a *Col2a1* intron 1 probe as a positive control, two *Ctgf2* promoter probes, Probes A and B, and mutated Probes A and B. The upper strands of the probes were as follows: *Col2a1* intron 1, 5′GGCGCTTGAGAAAAGCCCCATTCATGAGAGG 3′; Probe A, 5′ GGCCTGTTTGTGTAGGACTCCATTCAGTTCT 3′; Probe B, 5′ GGTTGTGTAGGACTCCATTCAGTTCTTTGGC 3′; mutated Probe A, 5′ GGCCTGTTTGTGTAGGACTCCAGCCAGTTCT 3′; and mutated Probe B, 5′ GGTTGTGTAGGACTCCATTCAGTTCTGCGGC 3′. The probes used for EMSA were labeled with α^32^P-dCTP using klenow fragment. EMSA was performed using truncated human recombinant SOX9 protein (a.a. 1–300), which was prepared as described below. Wild-type human *Sox9* DNA was digested with Sma1, which cut at 900 bp from the transcription start site. The resultant SOX9 fragment (a.a. 1–300) was cloned in frame into the histidine-tag–containing pET23 vector (EMD4 Biosciences (Novagene, Gibbstown, NJ)). The SOX9 fragment was overexpressed in *E. coli* BL21 cells and isolated from cell lysates with nickel resin (Qiagen, Valencia, CA) according to the manufacturer’s protocol.

The protein-DNA binding reactions were carried out with 5 fmol of ^32^P-end-labeled probe in a buffer containing 20 mM HEPES (pH 7.9), 50 mM KCl, 0.1% (v/v) Nonidet P-40, 0.5 mM EDTA, 40 mM dithiothreitol, 1 mM phenylmethyl sulfonyl fluoride, 30 μg of bovine serum albumin, and 20 ng of poly (dG-dC), with or without purified SOX9 (a.a. 1–300). The reaction mixtures (25 μl) were incubated for 10 min at 37 °C and electrophoresed on a 5% polyacrylamide gel in 0.5X Tris/Borate/EDTA buffer at 150 V for 2.5 h at room temperature. All experiments were performed three times, providing very similar results. Alternatively, EMSAs were performed using biotin 3′-end labeled probes for the SOX9 binding sites identified in nucleus pulposus nuclear extracts (Fig. 5). Probes were incubated with 1 μg of nuclear protein extracts for 20 minutes at room temperature following the LightShift Chemiluminescent EMSA Kit protocol (Thermo scientific).

### Generation of Sox9 conditional knockout mice

*Col2a1-CreER*^*T2*^ transgenic mice were bred with *Sox9*^*floxflox*^ mice (obtained from Jackson lab)^16^,^48^. Tamoxifen was injected to the control group *Sox9*^*floxflox*^ and the experimental group *Sox9*^*Col2ER*^ (1 mg/10 g body weight, five days) intraperitoneally at P20. Mice were sacrificed when they were 2-month-old. All experiments in this study were carried out with the recommendation of the Guide for the Care and Use of Laboratory Animals of the National Institute of Health, USA. The procedures were approved by the Institutional Animal Care and Use Committee (IACUC) of Rush University Medical Center (Chicago). All experimental methods and procedures were carried out in accordance with the approved guidelines.

### Immunohistochemistry

The knee joint tissue and disc tissue were dissected from *Sox9*^*floxflox*^ mice and *Sox9*^*Col2ER*^ mice. Samples were fixed in 10% formalin, decalcified in 14% EDTA, and embedded in paraffin. Paraffin sections were boiled for 15 min in citrate buffer to reverse cross-links and unmask epitopes. Sections were blocked with 5% goat serum for 1 h and incubated with anti-CCN2 (Abcam) or anti-SOX9 (Millipore) at a dilution of 1:200 overnight at 4 °C. The following day sections were incubated with secondary antibody (Alexa-555 goat anti-rabbit, Abcam) for 1 h at room temperature and were mounted with ProLong Gold Antifade Reagent with DAPI (Cell signaling).

### Statistical Analysis

The values are expressed as mean ± SEM. For the statistical analysis of the differences of independent-samples (n = 3), Student *t* test were performed as appropriate. In order to evaluate the temporal alterations of genes responsive to 4-OH-TM treatment, one-way analyses of variance (ANOVAs) were performed followed by post hoc Bonferroni’s test (n = 3). Significance was accepted at the 0.05 level of probablility (*p* < 0.05). SPSS 13.0 software was used in all analyses.

## Additional Information

**How to cite this article**: Oh, C.-d. *et al*. SOX9 directly Regulates CTGF/CCN2 Transcription in Growth Plate Chondrocytes and in Nucleus Pulposus Cells of Intervertebral Disc. *Sci. Rep.* **6**, 29916; doi: 10.1038/srep29916 (2016).

## Supplementary Material

Supplementary Information
