# Supplementary material for: SOX9 directly Regulates CTGF/CCN2 Transcription in Growth Plate Chondrocytes and in Nucleus Pulposus Cells of Intervertebral Disc
Source: Sci Rep. 2016 Jul 20;6:29916. doi: 10.1038/srep29916 (PMC4951750; doi:10.1038/srep29916)
Supplement: Supplementary Information [file srep29916-s1.pdf]

SOX9 directly Regulates CTGF/CCN2 Transcription in Growth Plate Chondrocytes and in Nucleus Pulposus Cells of Intervertebral Disc

Chun-do Oh[1,2*], Hideyo Yasuda[2,4*], Weiwei Zhao[1,3], Stephen P. Henry[2], Zhaoping Zhang[2], Ming Xue[1], Benoit de Crombrugghe[2], and Di Chen[1, §]

[1]Department of Biochemistry, Rush University Medical Center, Chicago, IL 60612, USA
[2]Department of Genetics, The University of Texas, M.D. Anderson Cancer Center, 1515 Holcombe Blvd., Houston, TX 77030, USA,
[3]Department of Orthopaedics & Traumatology, Li Ka Shing Faculty of Medicine, The University of Hong Kong, Hong Kong, China

[4]Present address: Mariabiotech Co. Ltd, 23 Nangye-ro 30-gil, Dongdaemun-gu, Seoul, 130-812, Korea

*Both authors were equally contributed in this work.

Running title: SOX9 regulates CTGF transcription in chondrocytes

[§] To whom correspondence should be addressed (Email: di_chen@rush.edu)

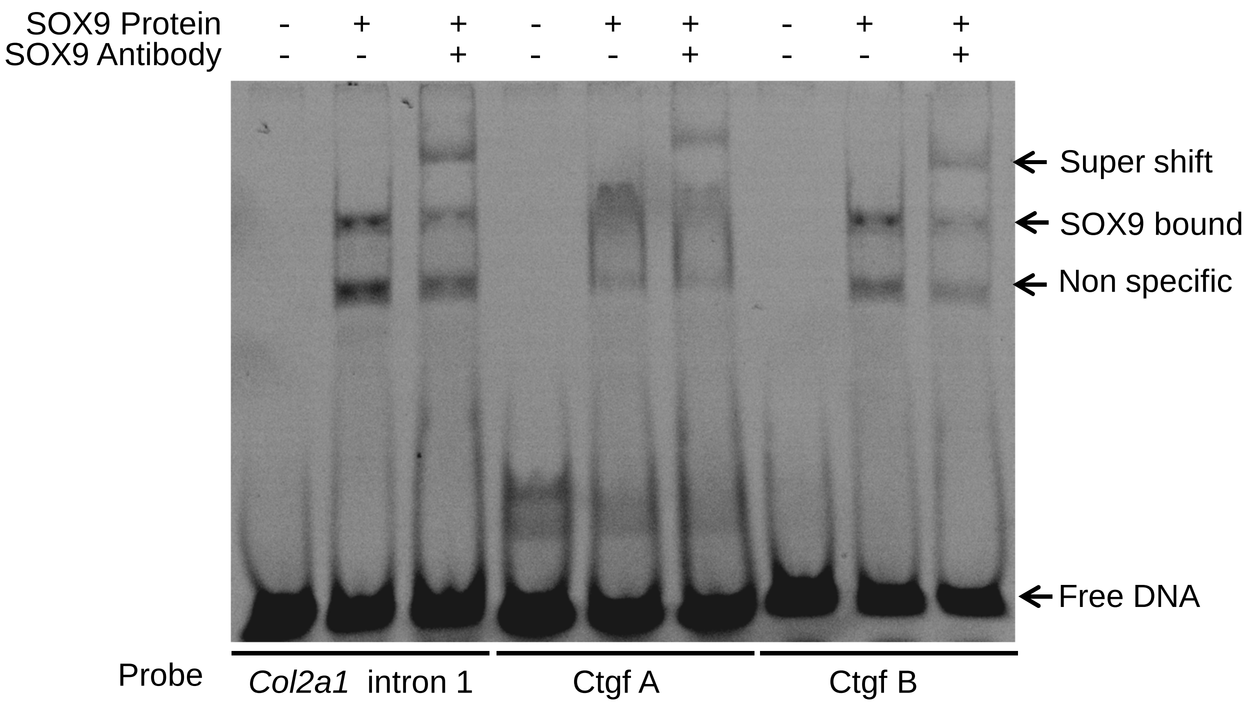

Fig. S1. Supershifts of the bands were observed with the addition of SOX9 antibody in EMSA assay. EMSA was performed as described in Methods with following minor modifications; here instead of labeling Col2a1-intron1-probe, ctgf A-probe and B-probe with 32P, they were labeled with Cy3 at 5' end (MacroGen, South Korea). In some reactions, 2mg of anti-SOX9 antibody (Cell signaling technology #82630, MA, USA) was added to the reaction. After the gel was run, the Cy3-labeled DNA was detected by KETA M series Imaging System (Wealtec Corp., NV, USA). All experiments were performed three times, providing similar results.

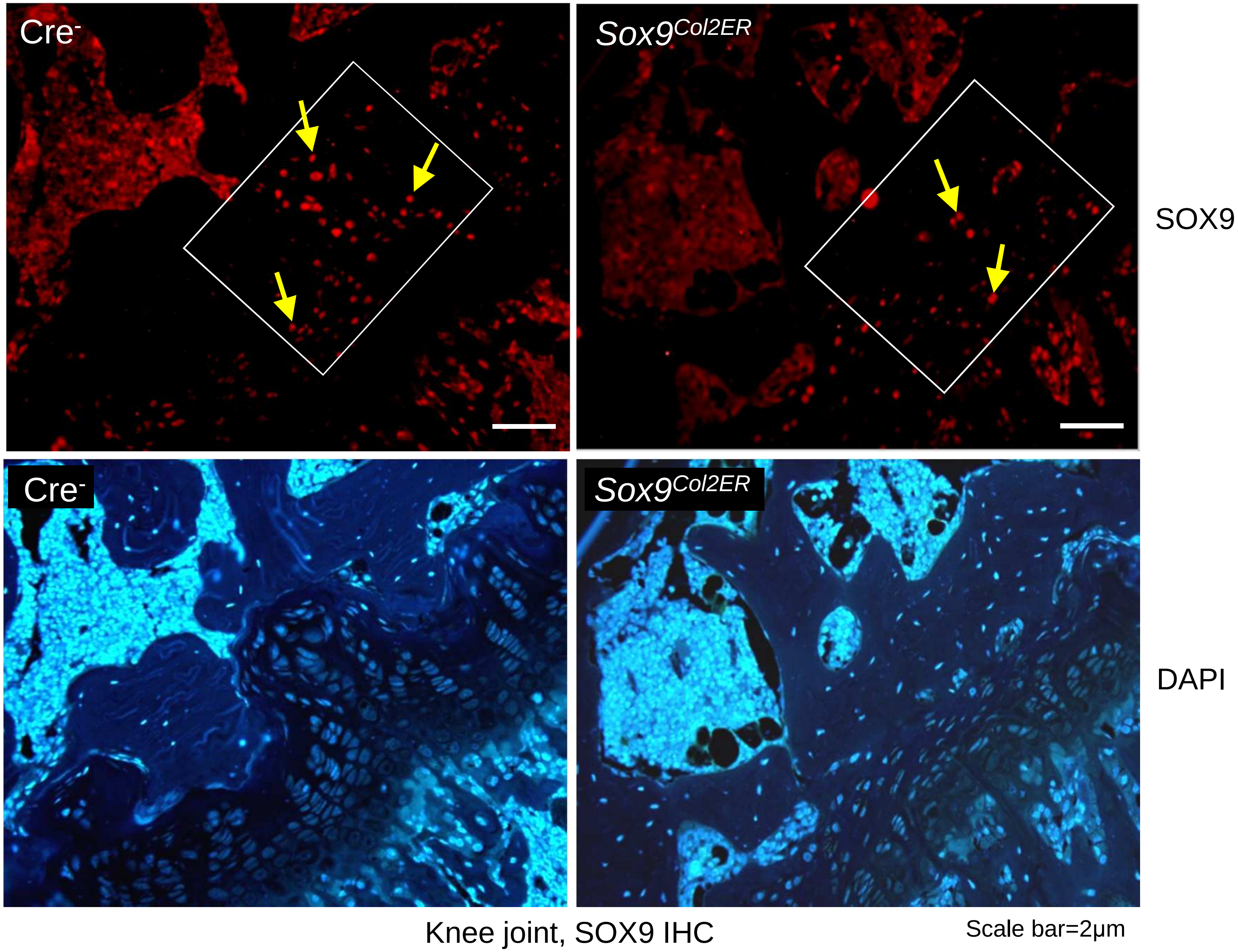

Fig. S2. SOX9 expression was reduced in growth plate cartilage of *Sox9^{Col2ER}* conditional knockout mice. SOX9 IHC was performed in histology sections from 2-month-old Cre^{-} and *Sox9^{Col2ER}* conditional knockout mice. Data showed that expression levels of SOX9 were significantly reduced in *Sox9^{Col2ER}* mice.

Gene	log2(FC)	% reduction
Sox9	-3.43	90.72
Ctgf	-1.47	63.90
Col2a1	-3.08	88.18

Fig. S3. RNA-Seq analysis. The total RNA was purified using the Trizol. The polyA-containing mRNA was prepared using oligo dT resin and then transcribed to cDNA using a cDNA preparation kit according to the manufacturer's protocol. After the adaptors were ligated to the cDNA, the cDNA library was prepared by PCR according to the manufacturer's protocol. The second-generation sequencing was performed from both ends by a Hi-Seq-2000 sequencer using the Illumina platform in the DNA Analysis Core facility at The University of Texas M.D. Anderson Cancer Center. The sequences obtained were aligned to the mouse genome sequence. For RNA-Seq, the RNA expression profile in primary *Sox9*$^{flox/flox}$ mouse chondrocytes infected with Ad-CMV-*Cre* was compared with that in the same cells infected with a control adenovirus. Analysis of RNA-Seq data indicates that the levels of *Sox9* mRNA were reduced 90% compared to the control levels by infection with Ad-CMV-*Cre.* Significant reduction in the levels of *Ctgf* and *Col2a1* was also observed as well.
